# Proteasome Inhibitors Block DNA Repair and Radiosensitize Non-Small Cell Lung Cancer

**DOI:** 10.1371/journal.pone.0073710

**Published:** 2013-09-05

**Authors:** Kyle R. Cron, Kaya Zhu, Deepa S. Kushwaha, Grace Hsieh, Dmitry Merzon, Jonathan Rameseder, Clark C. Chen, Alan D. D’Andrea, David Kozono

**Affiliations:** 1 Department of Radiation Oncology, Dana-Farber Cancer Institute, Boston, Massachusetts, United States of America; 2 Computational and Systems Biology, Massachusetts Institute of Technology, Cambridge, Massachusetts, United States of America; 3 Division of Neurosurgery, University of California San Diego, San Diego, California, United States of America; 4 Department of Pediatric Oncology, Dana-Farber Cancer Institute, Boston, Massachusetts, United States of America; Winship Cancer Institute of Emory University, United States of America

## Abstract

Despite optimal radiation therapy (RT), chemotherapy and/or surgery, a majority of patients with locally advanced non-small cell lung cancer (NSCLC) fail treatment. To identify novel gene targets for improved tumor control, we performed whole genome RNAi screens to identify knockdowns that most reproducibly increase NSCLC cytotoxicity. These screens identified several proteasome subunits among top hits, including the topmost hit *PSMA1*, a component of the core 20 S proteasome. Radiation and proteasome inhibition showed synergistic effects. Proteasome inhibition resulted in an 80–90% decrease in homologous recombination (HR), a 50% decrease in expression of NF-κB-inducible HR genes *BRCA1* and *FANCD2*, and a reduction of BRCA1, FANCD2 and RAD51 ionizing radiation-induced foci. IκBα RNAi knockdown rescued NSCLC radioresistance. Irradiation of mice with NCI-H460 xenografts after inducible *PSMA1* shRNA knockdown markedly increased murine survival compared to either treatment alone. Proteasome inhibition is a promising strategy for NSCLC radiosensitization via inhibition of NF-κB-mediated expression of Fanconi Anemia/HR DNA repair genes.

## Introduction

Radiation therapy (RT) is a critical modality in the treatment of lung cancer. It is highly effective when disease is localized, and curative doses can be safely administered. For example, Stage I non-small cell lung carcinomas (NSCLC) can be treated with sufficiently high doses of ionizing radiation (IR) to yield 3-year local control rates around 90% [1]. This is largely due to the high biologically effective doses (BED) that can be administered to isolated lung tumors using stereotactic body radiotherapy. Unfortunately, only 15% of patients present with such tumors [2]. More advanced tumors, which are significantly more common, cannot be treated to higher BED, due to infiltration of, or proximity to, radiosensitive structures including the lungs, spinal cord, esophagus and heart [3]. This limitation accounts for the higher local failure rates of around 30% [4]. Systemic agents are therefore administered to enhance the radiation response. Such agents include cytotoxic chemotherapeutics which themselves have significant dose-limiting toxicities and limited effects on tumor control. Only a minority of tumors exhibit genetic features, such as EGFR activating mutations [5] or EML4-ALK translocations [6], that allow for targeted therapies; additional options are needed.

Several genome-wide studies have been completed in NSCLC, including DNA sequencing [7], copy number analysis [8] and gene expression profiling [9]. These studies provided an unbiased identification of the most frequent and important genetic and epigenetic alterations among the 20–25,000 genes of the human genome [10]. They revealed intriguing associations among genes and clinical covariates, but were unable to directly demonstrate cause-and-effect relationships between alterations and therapeutic outcomes. In contrast, RNA interference (RNAi) can directly demonstrate the effects of reduced gene expression on cell physiology and survival [11].

Pooled short hairpin RNA (shRNA) screens, in which cancer cells are exposed to several thousand different shRNA sequences, averaging one gene knockdown per cell, have several compelling features. First, the effect of stable shRNA knockdown over several cell doublings can be explored, compared to transient transfection of small interfering RNA (siRNA) sequences with short-lived effects. Accordingly, shRNA expression mimics drug treatments that are typically given over several weeks rather than days. Also, cells harboring different shRNA sequences effectively compete with each other within the pool as they proliferate, giving rise to hits that yield more pronounced effects on proliferation [12].

Because radiotherapy is the mainstay of NSCLC treatment, gene silencings that result in synergistic cytotoxicity when combined with ionizing radiation (IR) are desirable. Many cancer treatments are additive in nature, and are combined because they result in differential side effect profiles [13]. Synergistic treatments that result in greater effects when administered concurrently, compared to the additive effects of each treatment given individually, may serve particularly well as radiosensitizers, since RT delivery may be constrained to a limited volume, and side effects may be less pronounced outside the irradiated volume.

Demonstrating the mechanism of a radiosensitizing shRNA may also reveal biomarkers for patient selection and treatment assessment. DNA double strand breaks (DSBs) are among the effects of IR that best correlate with its cytotoxicity. A single unrepaired DSB is sufficient to result in reproductive cell death via G2 arrest or mitotic catastrophe [14]. DSBs are predominately repaired through two pathways, homologous recombination (HR) and non-homologous end-joining (NHEJ) [15]. Gene silencings or small molecule inhibitors of either pathway may promote radiosensitization.

Here we examine proteasome inhibition as a strategy for NSCLC radiosensitization via inhibition of DNA DSB repair. Proteasome inhibition has been explored in multiple clinical trials enrolling NSCLC patients, with variable results. For example, a Phase II study of 114 patients treated with bortezomib plus gemcitabine and carboplatin as first-line treatment of advanced NSCLC, showed a response rate of 23% and a disease control rate (responses+stable disease) of 68%, thus warranting further studies [16]. Bortezomib showed no activity as monotherapy in a Phase II study of 14 patients, none of whom showed objective responses, and three (21%) had stable disease lasting 3.4–11.5 months [17]. Based on our data, we propose that proteasome inhibitors may be useful as radiosensitizers, given their repressive effects on NF-κB-mediated expression of genes required for the HR pathway.

## Results

### Multiple Proteasome Genes are Top Hits in NSCLC whole Genome RNAi Screens

A whole genome shRNA screen was conducted in two NSCLC cell lines, A549 and NCI-H460. These lines share common genetic features, including mutations in *KRAS* and *STK11* (aka LKB1). These mutations are found in tumors that are particularly aggressive and resistant to therapy, and for which targeted therapies are unavailable [18], [19]. The cell lines are wild-type in *TP53* and therefore less likely, compared to mutated lines, to exhibit genomic instability over the course of a screen spanning multiple weeks [20]. Also, their genetic similarity, in terms of key oncogenic and tumor suppressor genes, increases the probability that screen hits in one line will be reproduced in the other (Table S1).

**Figure 1:**
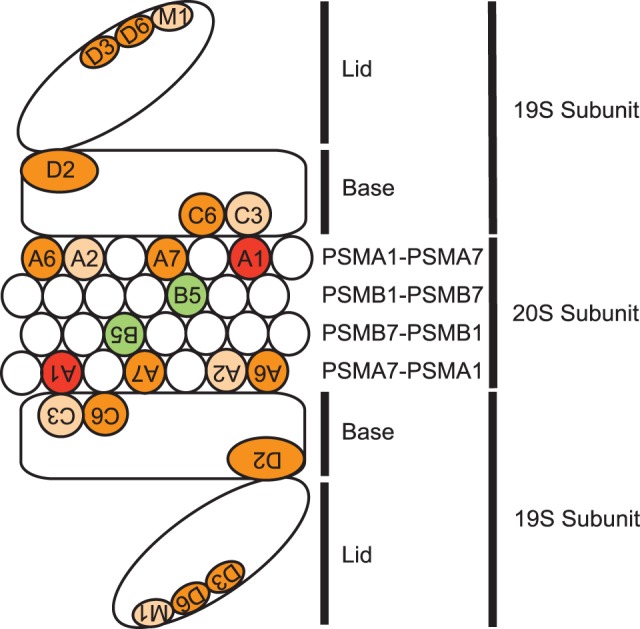
Knockdown of individual proteasome subunits results in NSCLC cytotoxicity. Diagram of the 26 S proteasome showing multiple whole genome shRNA screen hits with the following color code: top hit (red), strong hit (>1 shRNA sequence per gene in both cell lines, dark orange), minor hit (1 shRNA sequence per gene in both cell lines, light orange), chymotrypsin-like proteolytic catalytic site (not a hit but highlighted for illustrative purposes, green). Each hit is labeled using the last two alphanumeric characters of the gene’s HUGO nomenclature; e.g., A1 = PSMA1, B5 = PSMB5, M1 = SHFM1.

The Hannon-Elledge library contains 74,705 distinct shRNA sequences and targets nearly 18,000 genes [21]. After transduction with this library and puromycin selection for stable integrants, cells were passaged, and the relative representation of each sequence within a pool was determined before and after twelve population doublings. ShRNA sequences directed against genes essential for cellular proliferation were selectively lost. 1,667 genes were the targets of at least one shRNA sequence whose abundance decreased by at least two-fold during passage in both cell lines (Table S2). Multiple proteasome subunits numbered among the hits in both cell lines, including the top hit *PSMA1*, a subunit of the core 20 S proteasome [22] (Fig. 1 and Table S3).

### Proteasome Inhibitors Sensitize NSCLC Cells to Radiation

**Figure 2:**
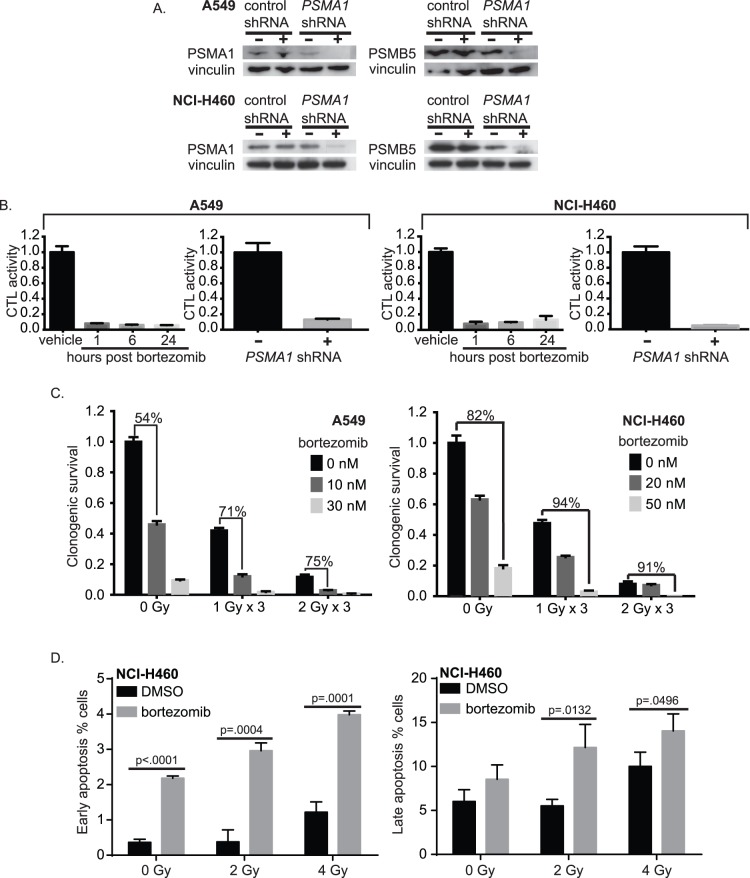
Proteasome inhibition sensitizes NSCLC cells to radiation. (A) Western blot showing protein levels of PSMA1 and PSMB5 in A549 and NCI-H460 NSCLC cells after *PSMA1* shRNA knockdown compared to non-silencing shRNA control. (B) Chymotrypsin-like (CTL) proteasome activity assay in A549 (left) and NCI-H460 (right) NSCLC cells after treatment with bortezomib, or *PSMA1* siRNA knockdown. All results are mean ± SEM and normalized to DMSO vehicle control. (C) Clonogenic survival assay of A549 (left) and NCI-H460 (right) following IR and bortezomib. Marked bars show the percent kill of bortezomib-treated samples compared to DMSO vehicle control at each IR dose. All results are mean ± SEM and normalized to DMSO vehicle control. (D) Apoptosis detection assay of NCI-H460 following 2 and 4 Gy IR and 50 nM bortezomib. Bars show percentage of cells in early apoptosis (left) or late apoptosis (right) via Annexin V and propidum iodide staining, respectively. All results are mean ± SD and P values were calculated using a two-tailed Student’s *t* test.

The doxycycline-inducible shRNA knockdown of *PSMA1* in A549 and NCI-H460 resulted in loss of protein expression of both PSMA1 and PSMB5, another subunit of the core 20 S proteasome and the catalytic site of chymotrypsin-like (CTL) activity of the proteasome [22] (Fig. 2A). This result confirms the essential role of *PSMA1* in 20 S proteasome assembly. Treatment with the small molecule proteasome inhibitor bortezomib or *PSMA1* shRNA knockdown caused a loss of CTL activity (Fig. 2B).

Bortezomib is an active agent *in vitro* in NSCLC lines, including A549 [23] and NCI-H460 [24], and it has previously demonstrated radiosensitizing properties in preclinical studies [25]. The ability of proteasome inhibitors to augment the effects of fractionated radiation, a clinical method of delivering daily radiation in small doses to minimize long-term toxicity [26], was therefore explored. Bortezomib and fractionated radiation yielded synergistic effects (Fig. 2C). Synergy was also observed with single-dose radiation (Fig. S1), although the result with fractionated radiation is more clinically relevant. This synergistic effect on cell death was mediated at least in part by apoptosis; 2 Gy IR alone did not induce apoptosis, while the combination of IR and bortezomib induced significantly increased apoptosis compared to either treatment alone (Fig. 2D).

### Proteasome Inhibition Impairs Radiation-induced DNA Double Strand Break Repair by Decreasing Homologous Recombination

**Figure 3:**
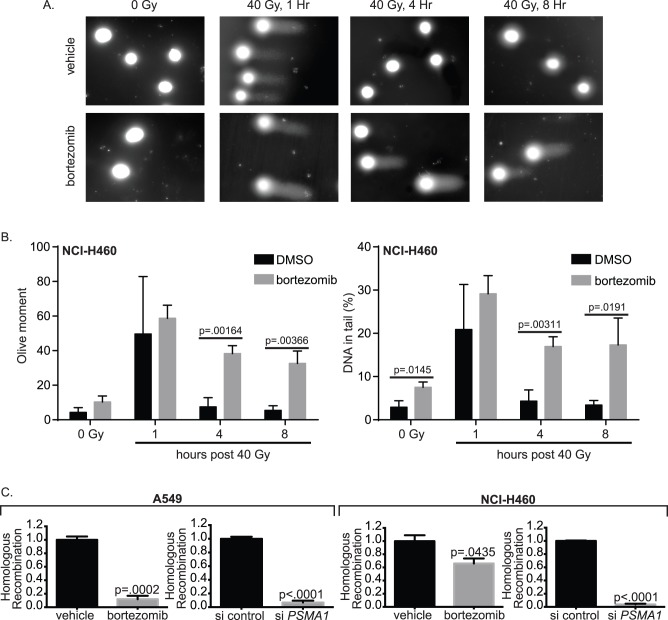
Proteasome inhibition impairs radiation-induced DNA double strand break (DSB) repair by decreasing homologous recombination. (A) Visualization of a neutral comet assay showing IR induced DNA DSB in NCI-H460 cells 1, 4 and 8 hours after 40 Gy IR and pre-treated with 50 nM bortezomib compared to DMSO vehicle control. (B) Quantification of the neutral comet assay via olive moment (left) and % DNA in tail (right) at 1, 4 and 8 hours post 40 Gy IR with and without 50 nM bortezomib in NCI-H460 cells. Each data point represents 3 independent replicate experiments of at least 50 cells. All results are mean ± SD and P values were calculated using a two-tailed Student’s *t* test. (B) GFP reporter assay for homologous recombination after proteasome inhibition for 24 hours via bortezomib or *PSMA1* siRNA knockdown in A549 (left) and NCI-H460 (right). All results are mean ± SD and normalized to DMSO vehicle control (Veh) or nonsilencing siRNA control. P values were calculated using a two-tailed Student’s *t* test.

Given the synergy between proteasome inhibition and radiation, we next determined whether proteasome inhibitors affect radiation-induced DNA double strand breaks (DSBs) [26] (Figure 3). In neutral comet assays, NSCLC cells repaired the vast majority of DNA DSBs generated by high-dose irradiation within one hour. In contrast, bortezomib treatment prior to radiation significantly delayed the repair of DNA DSBs at least up to 8 hours after irradiation (Fig. 3A–B). This persistence of DSBs may result in increased mitotic catastrophe [27], which is believed to be the predominant cause of cell death in irradiated solid malignancies.

To observe the effects of bortezomib or RNAi knockdown of *PSMA1* on DNA repair, a GFP reporter construct for HR [28] was introduced into A549 and NCI-H460 cells. Compared to untreated cells, those treated with either bortezomib or *PSMA1* siRNA showed a statistically significant decrease in HR-mediated repair of I-*Sce*I-induced DNA DSBs (Fig. 3C). Similar results were observed for NHEJ (Fig. S2). These results demonstrate a direct effect of proteasome inhibition on the mechanisms of DNA DSB repair, consistent with previous studies [29]–[31].

**Figure 4:**
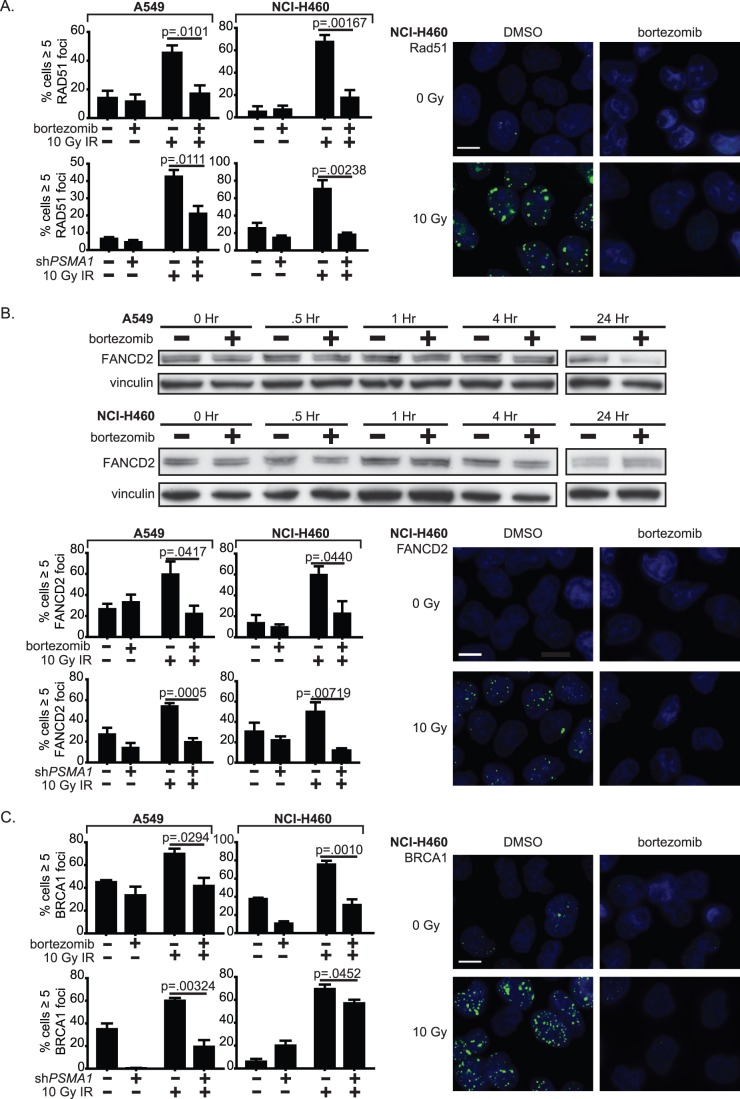
Proteasome inhibition impairs radiation-induced DNA double strand break repair by decreasing homologous recombination. (A) After bortezomib×24 hours or *PSMA1* knockdown×72 hours, A549 or NCI-H460 cells were irradiated then fixed after 6 hours. RAD51 foci were detected by immunofluorescence. Cells with ≥5 foci were scored as positive (n >100). All results are mean ± SEM. P values were calculated using a two-tailed Student’s t test. Photos show representative images for NCI-H460 treated with bortezomib, bar = 10 µm. (B) As in (A), but for FANCD2, including Western blot and immunofluorescence. (C) As in (A), but for BRCA1.

To understand the mechanism of HR inhibition, we explored the effect of bortezomib on nuclear focus formation of key proteins in the Fanconi Anemia (FA)/HR pathway. IR-induced RAD51 nuclear focus formation, which is a biomarker for HR [32], was substantially decreased by bortezomib or *PSMA1* RNAi (Fig. 4A, Fig. S3–S4). FANCD2 protein expression and IR-induced FANCD2 focus formation were similarly decreased by bortezomib or *PSMA1* RNAi (Fig. 4B, Fig. S3–S4). Finally, IR-induced BRCA1 focus formation was significantly decreased by bortezomib or *PSMA1* RNAi (Fig. 4C, Fig. S3–S4). Taken together, these data suggest that proteasome inhibition may impact HR-mediated repair of IR-induced DNA DSBs by reducing availability or recruitment of key FA/HR proteins to damage sites.

### Proteasome Inhibition Blocks NF-κB Induced Expression of Fanconi Anemia/Homologous Recombination Genes

**Figure 5:**
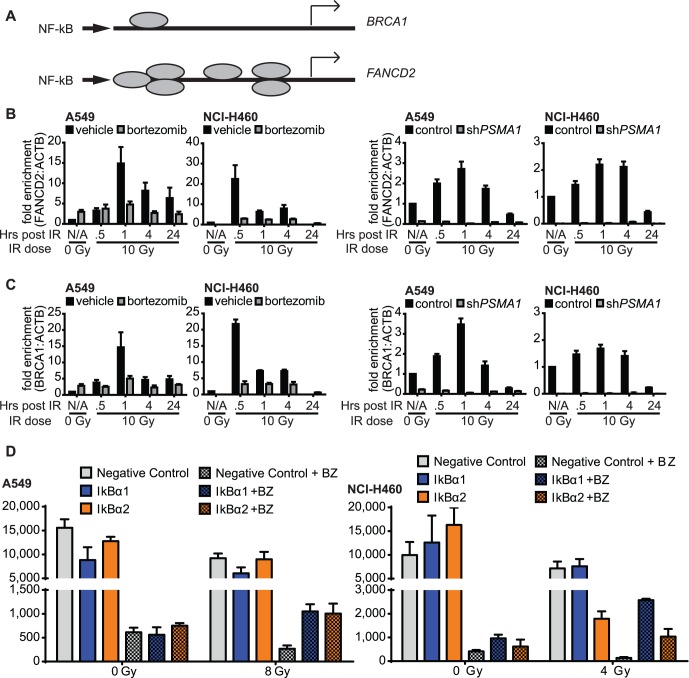
Proteasome inhibition blocks expression of Fanconi Anemia/Homologous Recombination genes. (A) Diagram of NF-kB promoters on *FANCD2* and *BRCA1* genes. (B) Expression by qPCR of *FANCD2* following proteasome inhibition ±30 nM (A549) or 50 nM (NCI-H460) bortezomib or ± inducible *PSMA1* shRNA, and ±10 Gy IR. All values are normalized to ACTB and all results are mean ± SEM. (C) As in (B) but with BRCA1. (D) NSCLC cell survival following bortezomib and IR is partially rescued by *IkBα* siRNA knockdown when performed in advance. Cell viability was assayed using an ATP luminescence-based assay measuring relative luminescent units (RLU). All results are mean ± SEM.

We next explored the relationship between proteasome inhibition and the FA/HR pathway via NF-κB signaling. Dalton and colleagues [33] previously showed that bortezomib decreases FA/BRCA gene expression in multiple myeloma cells, and that NF-κB transcriptionally upregulates the FA/BRCA pathway. For instance, NF-κB binds directly to the promoter of *FANCD2*, based on the electrophoretic mobility shift assay (EMSA) (Fig. 5A). One mechanism by which bortezomib downregulates the NF-κB pathway is through inhibition of proteasome-mediated degradation of IκBα [34]. IκBα, which sequesters NF-κB in the cytoplasm, and phosphorylation of IκBα by the inhibitor of NF-κB kinases (IKKs) releases NF-κB, allowing its entry to the nucleus [35]. Bortezomib also downregulates the NF-κB pathway by inducing nuclear translocation of IκBα, resulting in suppression of NF-κB p65/50-dependent transcription [36].

**Figure 6:**
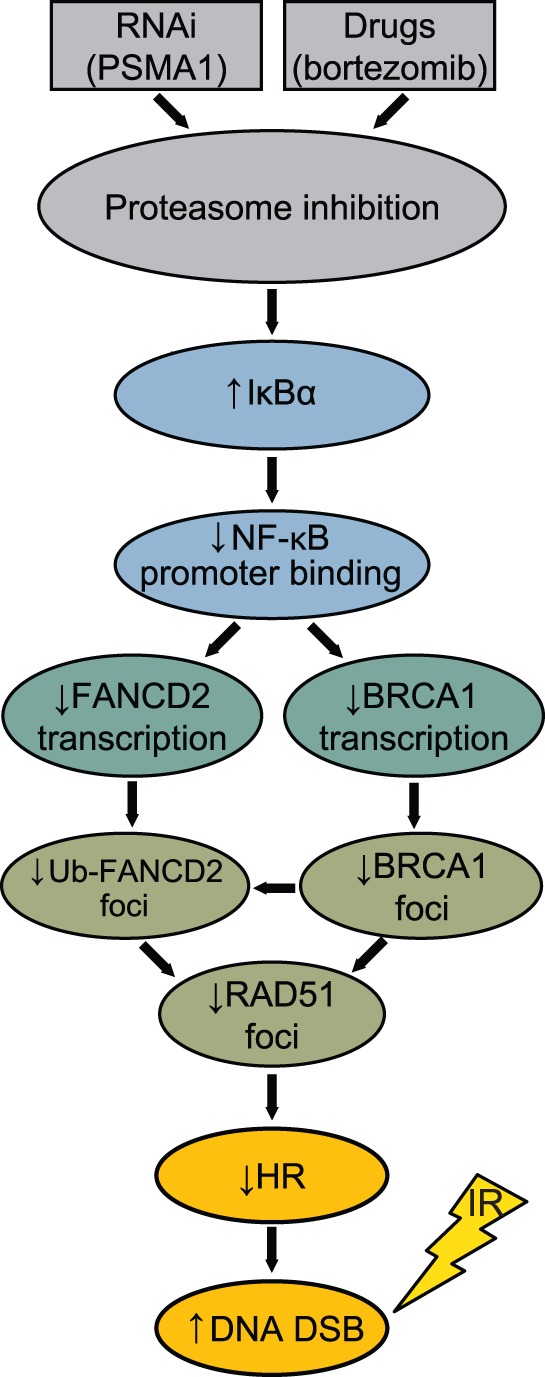
Role of proteasome inhibition in modulating NF-κB pathway-mediated expression of Fanconi Anemia (FA)/homologous recombination (HR) genes. Proteasome inhibition by bortezomib or *PSMA1* knockdown results in an increase in IκBα, which in turn decreases NF-κB binding to the promoters of FA/HR genes including *FANCD2* and *BRCA1*. This reduces the availability of these DNA repair proteins for recruitment to DNA damage sites, resulting in decreased RAD51 focus formation and HR following induction of DNA double strand breaks by ionizing radiation.

We therefore sought to determine whether the IR-induced transcription of FA/HR genes *FANCD2* and *BRCA1* is diminished by proteasome inhibition. Transcription of *FANCD2* and *BRCA1* increased within 30 minutes of irradiation, and was substantially blocked by either bortezomib or *PSMA1* RNAi (Fig. 5B–C). This short time scale could account for the short-term effect on DSB repair observed in the comet assay. To establish causality, we performed a rescue experiment in NSCLC cells pretreated with IκBα siRNA. Cells were then subjected to proteasome inhibition and irradiation. IkBa knockdown rescued radioresistance and cell survival of bortezomib-treated cells (Fig. 5D). These data establish a role for proteasome inhibitors as radiosensitizers, via blockade of NF-κB induced expression of FA/HR genes (Fig. 6).

### Combination of Proteasome Inhibition and Radiation Enhances Tumor Control

We next sought to determine whether proteasome inhibition may be a useful strategy *in vivo*. Poor penetration of bortezomib into tumors may limit its efficacy in solid malignancies [37]. We therefore tested whether inducible *PSMA1* RNAi knockdown enhances control of xenografts treated with fractionated radiotherapy (RT).

**Figure 7:**
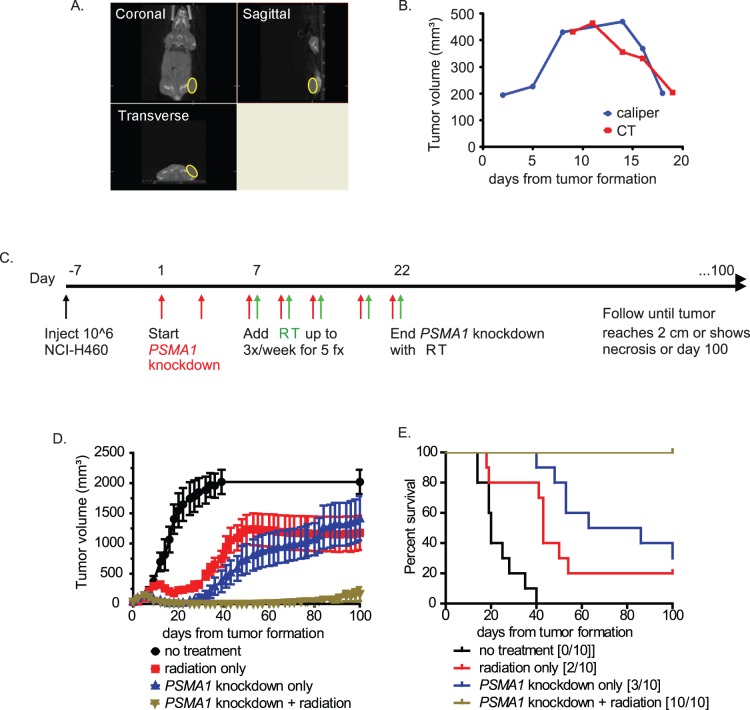
Combination of proteasome inhibition and radiation enhances tumor control. 10^6^ NCI-H460 cells transfected with doxycycline-inducible *PSMA1* shRNA were injected into the flanks of 6–8 week-old NCr nude mice. Once tumors reached 3 mm diameter (day 0), *PSMA1* knockdown was initiated with doxycycline drinking water. One week later, RT was initiated to give a total of five 4 Gy fractions every other day using a small animal radiation research platform (SARR). (A) Orthogonal images from a cone beam computed tomography (CT) scan, obtained using the SARRP, of a mouse bearing a subcutaneous xenograft. (B) Correlation of volumetric tumor measurements using the SARRP cone beam CT compared to traditional calipers. (C) Treatment schema. (D) Mice were subsequently followed until tumors reached 2 cm diameter, animals became moribund or for 100 days. (E) Kaplan-Meier analyses were performed with pairwise log-rank tests to assess differences in survival. Numbers surviving out of 10 mice per group on day 100 are indicated in brackets. For RT vs. RT+*PSMA1* knockdown, log rank *P = *0.0003.

To administer fractionated RT, we used a small animal radiation research platform (SARRP) that can deliver CT-guided conformal RT to tumors in mice [38], [39]. This platform ensures complete treatment of the tumor while minimizing irradiation of uninvolved tissues. It also facilitated volumetric assessments of tumor growth and partially correlated (r = 0.61) with caliper measurements (Fig. 7A–B).

**Figure 8:**
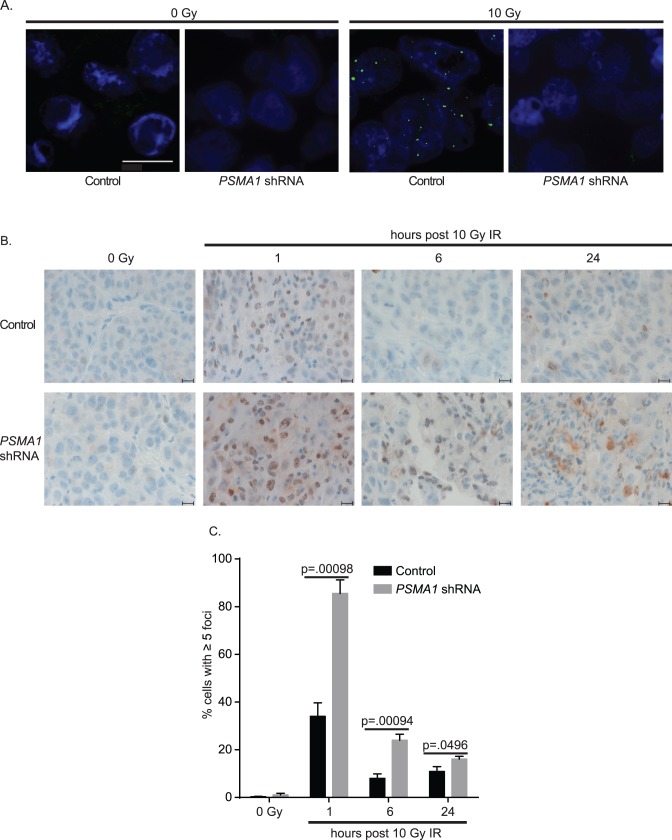
Proteasome inhibition delays DNA repair *in vivo*. (A) FANCD2 immunofluorescence in 10 Gy irradiated vs. unirradiated NCI-H460 xenografts recovered from mice with or without doxycycline-induced *PSMA1* shRNA expression in tumor cells. Bar = 10 µm. (B) γ-H2AX immunohistochemistry in NCI-H460 xenograft tumors with or without doxycycline-induced *PSMA1* shRNA knockdown, recovered from mice 1, 6, and 24 hours after 10 Gy irradiation. Bar = 10 µm. (C) Quantification of immunohistochemistry for γ-H2AX in (B). Cells with ≥5 foci were scored as positive (n>400 cells). All results are mean ± SEM. P values were calculated using a two-tailed Student’s t test.

Mice were implanted with NCI-H460 cells harboring a doxycycline-inducible *PSMA1* shRNA construct. One week after initiation of *PSMA1* knockdown, tumors were irradiated to a dose of 20 Gy in five fractions. After RT treatments, *PSMA1* knockdown was discontinued, and mice were followed for up to 100 days after tumor formation (Fig. 7C). Untreated mice showed rapid development of tumors. Mice treated with either RT or the inducible *PSMA1* knockdown in tumor cells also showed progressive tumor growth and poor survival. Mice treated concurrently with both treatments however showed minimal to no xenograft growth and 100% survival (Fig. 7D–E). IR-induced FANCD2 focus formation was also reduced in tumor specimens showing inducible *PSMA1* knockdown (Fig. 8A). The reduction in these foci may serve in the future as a biomarker for reduction of IR-induced activation of FA/HR pathway following proteasome inhibition. Significantly increased IR-induced γ-H2AX foci in tumor specimens persisted at 1, 6, and 24 hours with *PSMA1* knockdown compared to control, indicating delayed DNA DSB repair. (Fig. 8B–C).

The dramatic results observed with *PSMA1* knockdown are in contrast with those observed previously with bortezomib *in vivo*, including genetically engineered mouse models (GEMMs) of lung adenocarcinoma [40]. Given the results with *PSMA1* knockdown, the ability of bortezomib to radiosensitize NCI-H460 xenografts was examined to determine whether there may be greater tumoricidal effect with this combination therapy. There was no significant difference in survival of mice treated with radiotherapy with or without bortezomib (Figure S5). To determine whether this was due to poor tumor drug penetration, chymotrypsin-like proteasome activity was examined in tumors treated with or without bortezomib compared to tumors with or without PSMA1 knockdown. There was substantially more proteasome inhibition following PSMA1 knockdown compared to that following bortezomib (Figure S6). This suggests that the limited efficacy observed with bortezomib *in vivo* may be related to poor tumor drug penetration, which would explain the discrepancies in results *in vitro* and *in vivo*.

## Discussion

The purpose of this study was to establish a rationale for proteasome inhibition as a strategy for NSCLC radiosensitization. We reasoned that since DNA double strand breaks (DSBs) correlate with the cytotoxicity of ionizing radiation (IR) [26], agents that delay the repair of these breaks may act as radiosensitizers. Accordingly, we observed decreases in homologous recombination (HR) following proteasome inhibition, significant delays in DNA DSB repair both *in vitro* and *in vivo,* and reduced levels of IR-induced expression and damage site localization of DNA repair proteins. Proteasome inhibitors function, at least in part, by blocking the NF-κB pathway by interfering with degradation of IκBα, which inhibits downstream NF-κB activity [34], [35]. Bortezomib decreased the IR-induced expression following proteasome inhibition of Fanconi Anemia (FA)/BRCA genes, the majority of which have putative NF-κB binding sites [33]. Importantly, we rescued bortezomib-induced radiosensitivity by silencing IκBα, supporting the proposed mechanism. Proteasome inhibitors therefore appear to radiosensitize NSCLC by interfering with DNA DSB repair by reducing the expression of key NF-κB-inducible HR genes.

An alternate explanation for the effects of proteasome inhibition on radiosensitivity is through the induction of apoptosis. Multiple studies have demonstrated an increase in apoptosis following bortezomib treatment [41]. Apoptosis has not been shown as the predominant mode of cell death in irradiated NSCLC. For example, high doses such as 20 Gy irradiation of A549 or NCI-H460 cells yield rates of apoptosis of only 5–35% [42]. NCI-H460 cells treated with both 2 Gy IR and bortezomib showed increased apoptosis compared to either treatment alone. Of note, the persistence of DNA DSBs following irradiation of cells exposed to proteasome inhibitors may also itself contribute to apoptosis.

We observed reduced growth of NSCLC xenografts following proteasome inhibition combined with RT. GEMMs of lung cancer may be used to confirm the effect in primary lung tumors treated *in situ*. Exploring this in multiple GEMMs may have the additional advantage of identifying genotypes that particularly respond to proteasome inhibition. For instance, tumors arising from mice harboring inducible *KRAS* and *TP53* mutations responded to bortezomib, unlike tumors from mice with mutant *KRAS* and wild-type *TP53* [40]. We observed marked differences in survival with inducible proteasome knockdown in NSCLC xenografts expressing wild-type *TP53*, which is in contrast with these results. We propose that the combination with radiation may unlock the potential of proteasome inhibition to a wider range of tumor genotypes.

The adoption of proteasome inhibitors in the clinic will require improvement in tumor delivery. Bortezomib yielded relatively little proteasome inhibition in our xenograft studies compared to doxycycline-induced *PSMA1* shRNA knockdown. Strategies including liposomal encapsulation, which has been successfully employed for doxorubicin [43], or use of second-generation proteasome inhibitors such as carfilzomib, marizomib or MLN9708 [44] may improve solid tumor penetration and proteasome inhibition.

The results of proteasome inhibition in NSCLC patients treated without radiotherapy have been mixed [16], [17]. Proteasome inhibition was examined with concurrent chemoradiotherapy in one Phase I trial of twelve patients [45]. In this dose escalation study, patients with pathologically documented Stage IIIA-B disease received weekly carboplatin and paclitaxel, together with bortezomib 0.3–0.7 mg/m^2^ twice weekly, during radiotherapy to a dose of 61.2 Gy in 34 daily fractions, followed by surgical resection. There were no unanticipated acute toxicities during chemoradiotherapy; Grade 2–3 myelosuppression was common, as expected with carboplatin and paclitaxel. Regrettably however, three of nine patients who underwent surgical resection died postoperatively; two died two to three days postoperatively and a third died 21 days postoperatively. It was concluded that delayed toxicity was severe and unpredictable. However, all three patients had undergone a right pneumonectomy after high-dose neoadjuvant chemoradiotherapy. Right pneumonectomy following neoadjuvant therapy has been associated with significantly increased mortality risk, regardless of addition of novel systemic agents. For example, there has been a reported 18% treatment-related mortality rate after right pneumonectomy, compared to a 4% rate after left pneumonectomy, after neoadjuvant radiotherapy to a median dose of 54 Gy with concurrent chemotherapy [46].

What was notable about the Phase I study [45] was the high rate of pathologic complete response (pCR) observed in patients treated with neoadjuvant chemoradiotherapy including bortezomib. Specimens from five of the nine patients (56%) who underwent surgical resection showed a pCR and an additional two showed 99% necrosis. This compares favorably to the 17.7% pCR rate observed in INT-0139 following neoadjuvant chemoradiotherapy among the 164 patients who underwent resection [47]. This data suggests that, with care, proteasome inhibition may be worth exploring further combined with radical chemoradiotherapy, perhaps in nonsurgical candidates or for left-sided or other tumors that will not require right pneumonectomy for resection.

Finally, proteasome inhibitors may disproportionally benefit patients whose tumors rely on HR-mediated repair of radiation-induced DNA DSBs. For example, high level of RAD51 protein expression has been associated with decreased survival of NSCLC patients [48]. Also, poorly differentiated NSCLC tumors have increased expression of HR genes [49]. In an analysis of published microarray data from 442 patients [9], high levels of *RAD51* and *BRCA1* were each associated with decreased overall survival (Figure S7). These features may serve as biomarkers predictive of response to proteasome inhibitors as radiosensitizers. Also, reductions in radiation-induced DNA DSB repair protein foci, including FANCD2 and BRCA1, following treatment with proteasome inhibitors, may serve as biomarkers for response in treated tumors.

## Materials and Methods

### Reagents

Bortezomib was purchased from Selleck Chemicals (Houston, TX). 800 mM stocks in DMSO were stored at −20°C, and prior to use were diluted and stored at 4°C for up to one week. All treatments were at a final concentration of 30 nM for A549 or 50 nM for NCI-H460, unless stated otherwise. The following antibodies were used at the listed dilutions for Western immunoblots: PSMA1 (ARP40417, Aviva Systems Biology, San Diego, CA, 1∶2000), PSMB5 (BML-PW8895, Enzo Life Sciences, Farmingdale, NY, 1∶1000), FANCD2 (sc-20022, Santa Cruz Biotechnology, Santa Cruz, CA, 1∶200), vinculin (sc-25336, Santa Cruz Biotechnology, 1∶1000), anti-mouse (NA931v, GE Healthcare UK United, Little Chalfont, Buckinghamshire, UK, 1∶3000) and anti-rabbit (NA934v, GE Healthcare UK United, 1∶3000) horseradish peroxidase-linked secondary antibodies. The following antibodies were used at the listed dilutions for immunofluorescence: BRCA1 (sc-6954, Santa Cruz Biotechnology, 1∶50), RAD51 (PC-130, EMD Millipore, Billerica, MA, 1∶1000), FANCD2 (sc-20022, Santa Cruz Biotechnology, 1∶250), Alexa Fluor anti-mouse (4408 S, Cell Signaling Technology, 1∶3000) and anti-rabbit (4412 S, Cell Signaling Technology, 1∶3000).

### Cell Lines and Mice

Human NSCLC cell lines A549 and NCI-H460 were grown at 37°C in humidified 5% CO_2_ in Gibco RPMI 1640 (Life Technologies, Grand Island, NY) containing 10% fetal bovine serum (FBS, Sigma-Aldrich, St. Louis, MO) and 1 µg/ml Normocin (InvivoGen, San Diego, CA). HEK293T cells were grown in Gibco DMEM (Life Technologies) in 10% FBS and 1 µg/ml Normocin at 37°C in humidified 5% CO_2_. U2OS cells were grown in Gibco McCoy’s 5A (Life Technologies) in 10% FBS at 37°C in humidified 5% CO_2_. All cell lines were purchased from American Type Culture Collection (ATCC, Manassas, VA). 6–8 week old female homozygous CrTac:NCR-Foxn1 (nude) mice were purchased from Taconic Farms (Germantown, NY).

### Whole Genome Pooled shRNA Screen

The screen was performed essentially as previously described for DLD-1 cells [21]. The Hannon-Elledge whole genome pooled shRNA library consists of six viral pools each containing approximately 13,000 different MSCV-PM retroviral shRNA particles targeting human genes. For each pool, three replicates of at least 1.3×10^7^ cells of A549 or NCI-H460 cells were incubated with an equivalent number of retroviral colony-forming units in media containing 8 µg/ml polybrene (Sigma-Aldrich, St. Louis, MO), for a 1000-fold representation of each shRNA sequence at a multiplicity-of-infection (MOI) of 1. After selection for stable integrants using 1 µg/ml puromycin, cells were passaged for a total of twelve population doublings (PD), at all times maintaining a minimum of 1.3 × 10^7^ cells per replicate. Genomic DNA was extracted from cells harvested both before and after the twelve population doublings. Half-hairpin shRNA-containing sequences were amplified by PCR, purified by agarose gel electrophoresis and labeled with Cy5 (PD 0) or Cy3 (PD 12). Competitive hybridization to custom Agilent microarrays was performed.

For scoring, the mean log_2_ (Cy3/Cy5) ratio was determined for each shRNA sequence using triplicate data. Those shRNAs for which the standard deviation of log_2_ (Cy3/Cy5) ratios was greater than the absolute mean log_2_ (Cy3/Cy5) ratio were excluded from further analyses to reduce false positive hits. For each gene, one point was assigned for each different shRNA sequence targeting that gene for which the mean log_2_ (Cy3/Cy5) ratio was less than or equal to −1, representing a decrease in representation of the shRNA sequence of at least 2-fold during passaging. One half point was assigned for each shRNA sequence for which the mean log_2_ (Cy3/Cy5) ratio was less than or equal to −0.5. A negative point was assigned for each shRNA sequence for which the mean log_2_ (Cy3/Cy5) ratio was greater than or equal to +1, and a negative half point was assigned for each shRNA sequence for which the mean log_2_ (Cy3/Cy5) ratio was greater than or equal to +0.5, to penalize discordant results among shRNA sequences targeting a particular gene. Finally, genes were ranked based on descending total score, with higher rankings given to genes with equivalent total scores but fewer different shRNA sequences targeting the gene.

### PSMA1 shRNA Inducible Cell Lines

The TRIPZ plasmid containing *PSMA1* shRNA sequence V2THS_170669 was purchased from Thermo Scientific (Rockford, IL). The non-silencing shRNA control (RHS4743, Thermo Scientific) was also purchased. Lentiviral particles were generated using the Trans-Lentiviral Packaging Mix (Thermo Scientific) in HEK293T cells per manufacturer’s protocol. Viral titers were assessed by serial dilution using U2OS cells. A549 and NCI-H460 cells were infected at a MOI of 1.5. Transfected cells were selected with puromycin, and individual subclones were tested for doxycycline-inducible *PSMA1* shRNA knockdown via Western blot.

### Proteasome Activity Assay

96-well white, clear bottom polystyrene tissue culture plates (#3903, Corning, Tewksbury, MA) were seeded with 1000 NSCLC cells in 200 µl media. After 24 hours, media was replaced with media containing bortezomib, or *PSMA1* shRNA was induced with 1 µg/ml doxycycline. Cells were incubated for up to 3 days prior to chymotrypsin-like proteasome activity assays using the Proteasome-Glo cell-based luminescent assay (Promega, Madison, WI) per manufacturer’s protocol. All results were normalized to cells treated with DMSO vehicle control for bortezomib or non-silencing control for RNAi knockdown.

### Clonogenic Cell Survival Assay

NSCLC cells were seeded into 6-well tissue culture-treated dishes (#3516, Corning, Tewksbury, MA) at 10% confluence. Bortezomib was initiated 24 hours after seeding and continued during IR initiated 24 hours after bortezomib. IR was given using a Gammacell 40 Exactor Cs-137 irradiator (Best Theratronics, Ottawa, Ontario, Canada) in 1 or 2 Gy daily fractions over three days. Six hours after the final fraction, cells were trypsinized, counted, and seeded into 10 cm dishes in triplicate to generate isolated colonies. Once colonies became visible by eye, plates were stained with 1% crystal violet (Sigma-Aldrich) and colonies of at least 50 cells were counted by eye.

### Apoptosis Assay

NCI-H460 cells were seeded into 6-well tissue culture treated dishes (Corning) at 10% confluence. Bortezomib was initiated 24 hours after seeding and continued during IR. 2 to 4 Gy IR was administered 24 hours later using a Gammacell 40 Exactor Cs-137 irradiator (Best Theratronics). Cells were prepped and stained 24 hours later with the FITC Annexin V Apoptosis Detection Kit II (#556570, BD Biosciences, San Jose, CA) as per manufacturer’s protocol. Quantification was performed by flow cytometry (BD LSRFortessa, BD Biosciences, San Jose, CA).

### Western Immunoblots

For whole cell lysates, 1 ml ice-cold RIPA buffer [proteasome inhibitor Complete mini (11 836 153 001, Roche Diagnostics, Mannheim, Germany), 150 mM NaCl (Sigma-Aldrich), 1.0% IGEPAL CA-630 (Sigma-Aldrich), 0.5% sodium deoxycholate (Sigma-Aldrich).01% SDS (Bio-Rad, Hercules, CA), 50 mM Tris, pH 8.0 (Bio-Rad)] per 10^7^ cells was used. Semi-dry transfer was performed using the Trans-Blot SD apparatus onto PVDF membrane (Bio-Rad) per manufacturer’s protocol. Western Lightning Plus enhanced chemiluminesence substrate (PerkinElmer, Waltham, MA) was used for visualization on Amersham Hyperfilm (GE Healthcare, Pittsburgh, PA).

### Immunofluorescence

Cells were fixed with 4% formaldehyde (Sigma-Aldrich) followed by ice-cold methanol 6 hours post IR. Slides were incubated on a rocker at room temperature for 2 hours in blocking buffer containing 10% normal goat serum and 0.3% Triton X-100, then incubated in primary antibody overnight followed by secondary antibody for one hour in antibody buffer containing PBS, 1% bovine serum albumin and 0.3% Triton X-100). ProLong Gold antifade reagent with DAPI (Life Technologies, Grand Island, NY) was used for mounting. An Axio Imager Z1 fluorescence microscope with AxioCam MRc and MRm CCD cameras (Zeiss, Thornwood, NY) was used for visualization. DNA damage-inducible foci were counted manually in 100 cells per sample, with a minimum of 3 replicates.

### Comet Assay

Cells were seeded into 6-well plates at 20% confluence. Bortezomib was initiated 24 hours after seeding and refreshed 1 hour prior to IR the following day. 40 Gy IR was given using a Gammacell 40 Exactor with cells on ice, and cells were collected 1, 4 or 8 hours after initiation of IR. The OxiSelect Comet Assay Kit (Cell Biolabs, San Diego, CA) was used per manufacturer’s protocol, and comets were visualized using an Axio Imager Z1 fluorescence microscope with an AxioCam MRm CCD camera (Zeiss, Thornwood, NY).

### Homologous Recombination (HR) and Non-homologous End-joining (NHEJ) Assays

Plasmids phprtDRGFP, pCBA*Sce* [28] and pEJ [50] were a gift from Dr. David Weinstock. A549 and NCI-H460 cells were transfected with phprtDRGFP (for HR assays) or pEJ (for NHEJ assays) via FuGENE HD (Roche, Indianapolis, IN). Subclones harboring single stable integrations were selected by Southern blot as previously described [28]. GFP reporter assays were performed as previously described [28], [50]. Cells were transiently transfected with pCBA*Sce* to induce DNA double strand breaks at I-*Sce*I sites introduced via phprtDRGFP or pEJ. Cells were treated with bortezomib starting 24 hours before pCBA*Sce* transfection, or transfected with 25 nM siGENOME *PSMA1* siRNA (D-010123-05, Thermo Scientific) or AllStars Negative Control siRNA (Qiagen, Valencia, CA) via Lipofectamine RNAiMAX reagent (Invitrogen, Carlsbad, CA) 24–48 hours before pCBA*Sce* transfection. After pCBA*Sce* transfection, cells were incubated 24–96 hours prior to quantification of GFP-expressing cells by flow cytometry (BD LSRFortessa, BD Biosciences, San Jose, CA).

### Quantitative RT-PCR

Total RNA was isolated using the RNeasy mini kit (Qiagen) per manufacturer’s protocol. cDNA was synthesized using the Iscript cDNA kit (BioRad, Hercules, CA) per manufacturer’s protocol. Quantitative RT-PCR was performed using *BRCA1* and *FANCD2* intron-spanning primers (Eurofins MWG Operon, Huntsville, AL) with SYBR Green (Bio-Rad) master mix; *BRCA1*: CAACATGCCCACAGATCAAC (forward) and ATGGAAGCCATTGTCCTCTG (reverse), FANCD2: ACGGTGCTAGAGAGCTGCTT (forward) and TGTTCTCAGCACACTGGCAT (reverse). Data was normalized using *ACTB*: TGAAGTGTGACGTGGACATC (forward) and GGAGGAGCAATGATCTTGAT (reverse).

### IκBα Rescue

A549 or NCI-H460 cells were seeded into 6-well tissue culture dishes and then transfected with 25 nM IκBα siRNA (#1 = si00126826, #2 = si03114630, Qiagen) or AllStars Negative Control siRNA (Qiagen). After 24 hours, bortezomib was added, and then 24 hours later, cells were treated with 10 Gy IR. Cells were assayed for viability 24 hours later by the CellTiter-Glo assay (Promega, Madison, WI).

### Mouse Xenograft Experiments

NCI-H460 cells were trypsinized and suspended at 5×10^6^ cells/ml in 0.9% saline. 1×10^6^ cells were injected subcutaneously into the right flanks of NCr nude mice. Tumor formation was assessed by caliper, with tumor volume determined by 0.5×longest diameter×shortest diameter^2^. Once tumors reached at least 200 mm^3^, *PSMA1* shRNA knockdown was induced by including 0.5 mg/ml doxycycline in drinking water. In a separate cohort, 0.6 mg/kg bortezomib given intravenously by tail vein was started. One week later, IR was initiated to give five fractions of 4 Gy up to three times per week using a small animal radiation research platform (SARRP) as described below. In the bortezomib cohort, treatment was administered 1 hour prior to RT. Doxycycline or bortezomib was discontinued after the final fraction of RT. Tumors were assessed until they reached 2 cm diameter or showed necrosis, or mice showed evidence of morbidity, or 100 days after initiation of treatment, whichever came first. Mice were euthanized with carbon dioxide. For immunofluorescence, tumor samples were collected immediately and embedded in O.C.T. (VWR, 4583) in Tissue-tek cryomold (VWR, 4557) plastic cassettes. Cassettes with sample and O.C.T. were then placed above liquid nitrogen until frozen and stored at −80°C. For immunohistochemistry, tumor samples were collected and fixed in 10% formalin acetate (SF99-4, Fischer Scientific) for 24 hours then stored in 70% ethanol prior to paraffin embedding and sectioning. γ-H2AX (05–636, Millipore, Billerica, MA) staining at a dilution of 1∶20,000 was performed by the Dana-Farber/Harvard Cancer Center Research Pathology Core. An Axio Imager Z1 fluorescence microscope with an AxioCam MRc camera (Zeiss) was used for visualization. γ-H2AX foci were counted manually with a minimum of 400 cells per sample. For proteasome activity assays, tumor samples were collected immediately following euthanasia and snap frozen and stored at −80°C. PBS-EDTA buffer was then added and samples sonicated for 60 seconds. Proteasomal activity of the lysate was then assessed using the Proteasome-Glo assay (Promega) per manufacturer’s protocol.

### Small Animal Radiation Research Platform (SARRP)

Mice were anesthetized via isoflurane inhalation for the duration of each treatment. For each treatment, tumors were visualized using cone beam computed tomography (CT) using 65 kVp and 0.6 mA photons. Tumors were treated using a 1.2 cm circular collimator selected to give 0.25–0.5 cm margins around gross tumor, using 220 kVp and 13 mA photons given with a lateral en face field prescribed to a depth of 5 mm. The SARRP was calibrated and maintained as previously described [38], [39].

### Bioinformatics

Affymetrix HG-U133A CEL files and clinical annotation data from a published microarray dataset [9] were downloaded from https://array.nci.nih.gov/caarray/project/details.action?project.experiment.publicIdentifier=jacob-00182. Gene expression summary values were computed using the Robust Multichip Average (RMA) [51] method using RMAExpress 1.0.5 obtained from http://rmaexpress.bmbolstad.com/, using an Entrez gene-centric custom CDF downloaded from http://brainarray.mbni.med.umich.edu/Brainarray/Database/CustomCDF/genomic_curated_CDF.asp. Kaplan-Meier survival analysis was performed for *BRCA1* and *RAD51* using SAS JMP 10 Pro (SAS, Cary, NC). Data on *FANCD2* was not available in this dataset.

### Statistics

All quantitative data were analyzed and graphs formed using Prism 6 (Graphpad Software, La Jolla, CA). All differences between treatment groups were determined via two-tailed parametric Student’s *t* test, with a *p* value <0.05 viewed as significant.

### Declaration of Ethical Approval

All animal experiments were performed in accordance with a protocol (11-024) approved by the Dana-Farber Cancer Institute Animal Care and Use Committee (DFCI ACUC) and in adherence to the NIH Guide for the Care and Use of Laboratory Animals.

## Supporting Information

Figure S1 **Clonogenic survival assay of A549 (left) and NCI-H460 (right) following single fraction IR and bortezomib.** Marked bars show the percent kill of bortezomib-treated samples compared to DMSO vehicle control at each IR dose. All results are mean ± SEM and normalized to DMSO vehicle control.
(EPS)

Figure S2 **Proteasome inhibition reduces non-homologous end joining in NSCLC cells.** GFP reporter assay for non-homologous end joining (NHEJ) after proteasome inhibition for 24 hours via bortezomib (right) or *PSMA1* siRNA knockdown (left) in A549 (top) and NCI-H460 (bottom). All results are mean ± SD and normalized to DMSO vehicle control (Veh) or scrambled vector control. P values were calculated using a two-tailed Student’s *t* test.
(TIF)

Figure S3 **Bortezomib reduces RAD51, FANCD2 and BRCA1 IR induced foci in A549.** These photos show representative images for the quantified data in Figure 4 of the main text; refer to that figure legend for additional details. Bar = 10 µm.
(TIF)

Figure S4 ***PSMA1*** **shRNA reduces RAD51, FANCD2 and BRCA1 IR induced foci in A549 and NCI-H460.** These photos show representative images for the quantified data in Figure 4 of the main text; refer to that figure legend for additional details. Bar = 10 µm.
(TIFF)

Figure S5 **Bortezomib does not improve control of irradiated NSCLC xenografts.** 10^6^ NCI-H460 cells were injected into the flanks of 6–8 week-old NCr nude mice. Once tumors reached 3 mm diameter (day 0), bortezomib was injected into the tail vein at 0.6 mg/kg twice per week. One week later, RT was initiated to give a total of five 4 Gy fractions every other day using a small animal radiation research platform (SARRP) with 0.6 mg/kg bortezomib given 1 hour prior to RT. Mice were subsequently followed until tumors reached 2 cm diameter, animals became moribund or for 100 days.
(TIF)

Figure S6 **Chymotrypsin-like proteasome activity in NSCLC xenografts following bortezomib vs. inducible** ***PSMA1*** **shRNA knockdown.** Tumors were collected and frozen 1 hour after the last of three doses of bortezomib given over one week, or proteasome inhibition via PSMA1 shRNA knockdown for one week. Control mice were treated with DMSO (Veh) or non-silencing shRNA tumors. All results are mean ± SEM.
(EPS)

Figure S7 **High** ***BRCA1*** **and** ***RAD51*** **gene expression are each associated with decreased overall survival.** Kaplan-Meier analysis was performed using a published dataset of 442 lung adenocarcinomas [9], comparing survival among patients with greater than median expression versus less than median expression of each gene. The log-rank test was performed to assess for statistically significant differences in overall survival.
(TIF)

Table S1 **Mutations in NSCLC cell lines.** List of sequenced DNA mutations in the NSCLC cell lines A549 and NCI-H460 from the Wellcome Trust Sanger Institute Cancer Cell Line project (http://www.sanger.ac.uk/genetics/CGP/CellLines/). A549 and NCI-H460 harbor mutations in the majority of the same oncogenic and tumor suppressor genes, including *CDKN2A*, *KRAS* and *STK11*.
(XLSX)

Table S2 **Whole genome shRNA screen hits in A549 and NCI-H460.** Complete list of whole genome shRNA screen hits in A549 and NCI-H460. Each of the 1,667 genes was the target of at least one shRNA sequence whose representation decreased by at least two-fold during passage in both cell lines. Listed are the numbers of shRNA sequences in the screen targeting each gene, and the score for each cell line. To derive the score, one point was given for each shRNA sequence whose representation decreased by at least two-fold during passage, and one half point was given for each sequence whose representation decreased by 2^0.5 to 1^-fold. One point was subtracted for each shRNA sequence whose representation increased by at least two-fold during passage, and one half point was given for each sequence whose representation increased by 2^0.5 to 1^-fold, to penalize discordant effects.
(XLSX)

Table S3 **List of 26 S proteasome genes highlighting whole genome shRNA screen hits**. Each of the highlighted genes was the target of at least one shRNA sequence whose representation decreased by at least two-fold during passage in both cell lines, with the top hit *PSMA1* shaded red, strong hits (>1 shRNA sequence per gene in both cell lines) shaded dark orange and minor hits (1 shRNA sequence per gene in both cell lines) shaded light orange. Listed are the numbers of shRNA sequences in the screen targeting each gene, and the score for each cell line. To derive the score, one point was given for each shRNA sequence whose representation decreased by at least two-fold during passage, and one half point was given for each sequence whose representation decreased by 2^0.5 to 1^-fold. One point was subtracted for each shRNA sequence whose representation increased by at least two-fold during passage, and one half point was given for each sequence whose representation increased by 2^0.5 to 1^-fold, to penalize discordant effects.
(XLSX)
