# Supplementary figures and images for: Proteasome Inhibitors Block DNA Repair and Radiosensitize Non-Small Cell Lung Cancer
Source: PLoS One. 2013 Sep 5;8(9):e73710. doi: 10.1371/journal.pone.0073710 (PMC3764058; doi:10.1371/journal.pone.0073710)

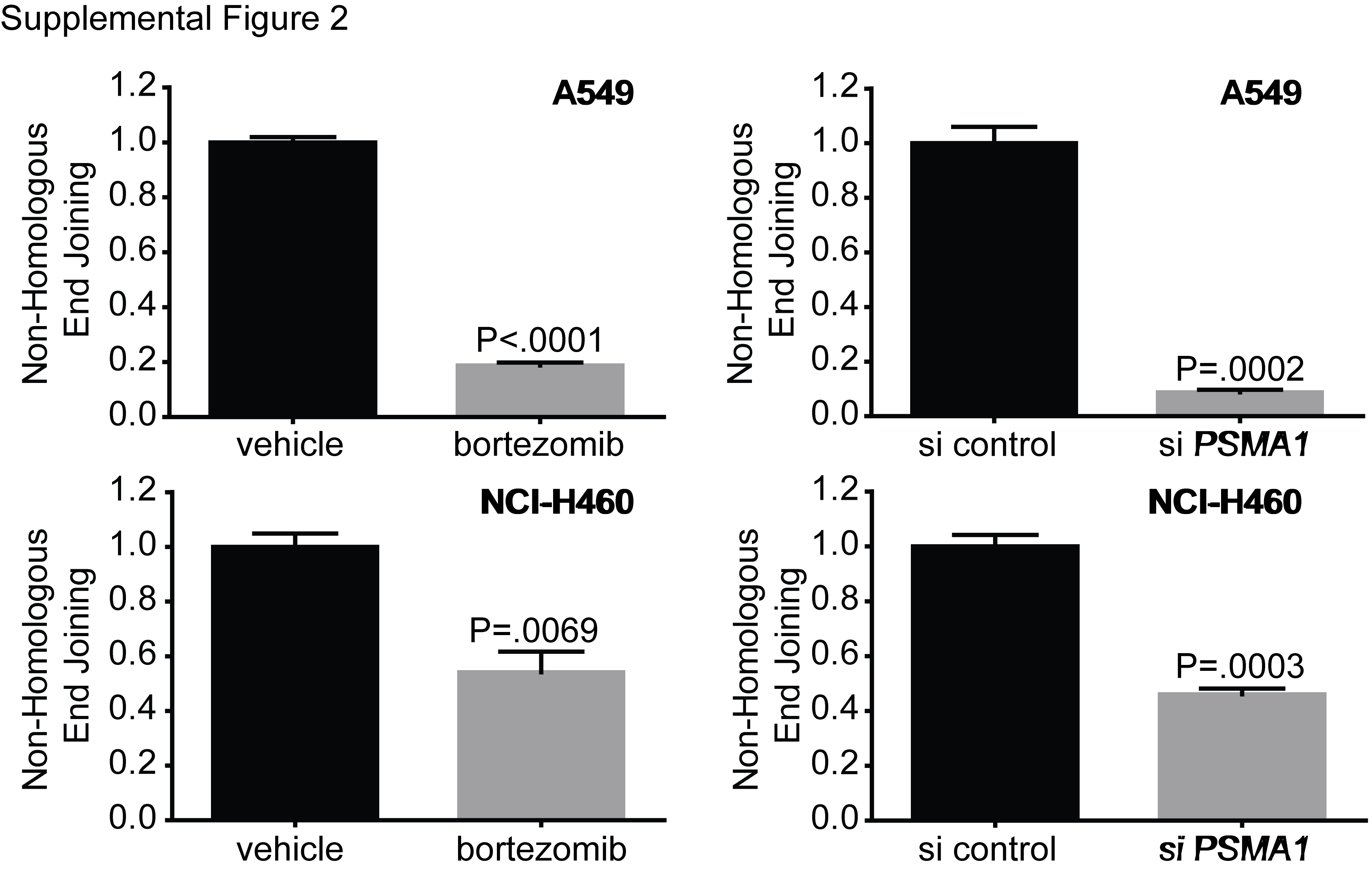

Supplement: Figure S2 — Proteasome inhibition reduces non-homologous end joining in NSCLC cells. GFP reporter assay for non-homologous end joining (NHEJ) after proteasome inhibition for 24 hours via bortezomib (right) or PSMA1 siRNA knockdown (left) in A549 (top) and NCI-H460 (bottom). All results are mean ± SD and normalized to DMSO vehicle control (Veh) or scrambled vector control. P values were calculated using a two-tailed Student’s t test. (TIF) [file pone.0073710.s002.tif]

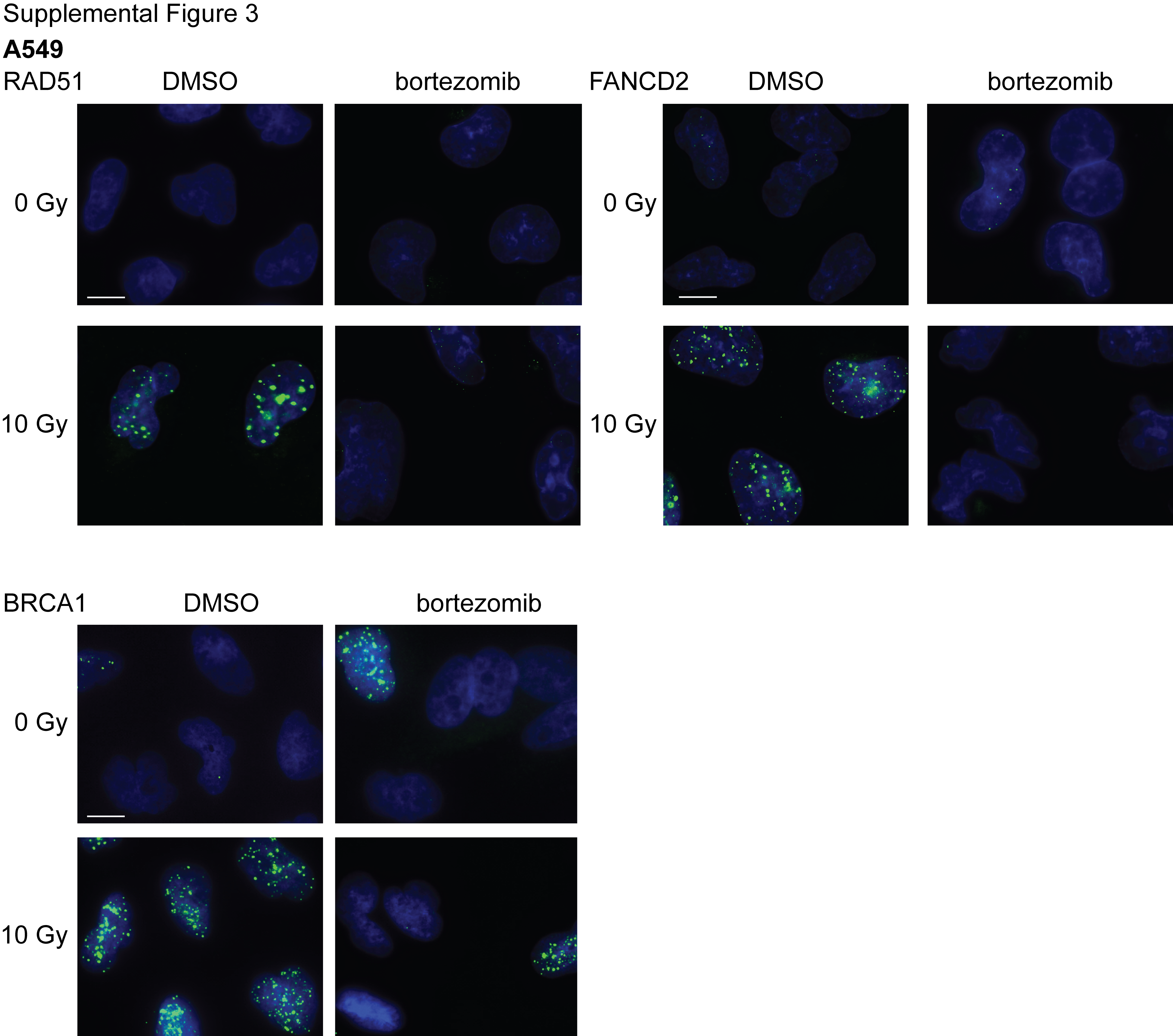

Supplement: Figure S3 — Bortezomib reduces RAD51, FANCD2 and BRCA1 IR induced foci in A549. These photos show representative images for the quantified data in Figure 4 of the main text; refer to that figure legend for additional details. Bar = 10 µm. (TIF) [file pone.0073710.s003.tif]

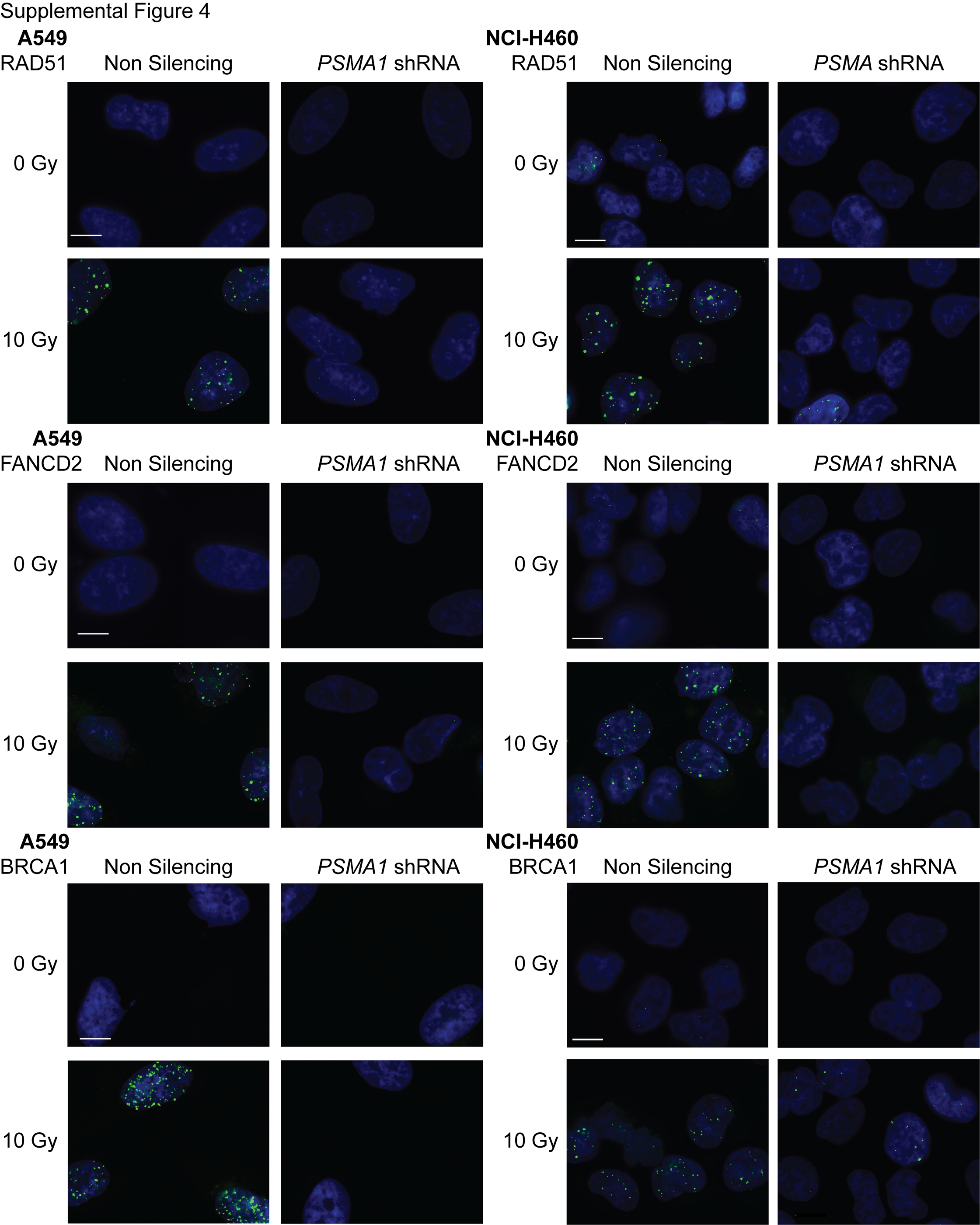

Supplement: Figure S4 — PSMA1 shRNA reduces RAD51, FANCD2 and BRCA1 IR induced foci in A549 and NCI-H460. These photos show representative images for the quantified data in Figure 4 of the main text; refer to that figure legend for additional details. Bar = 10 µm. (TIFF) [file pone.0073710.s004.tiff]

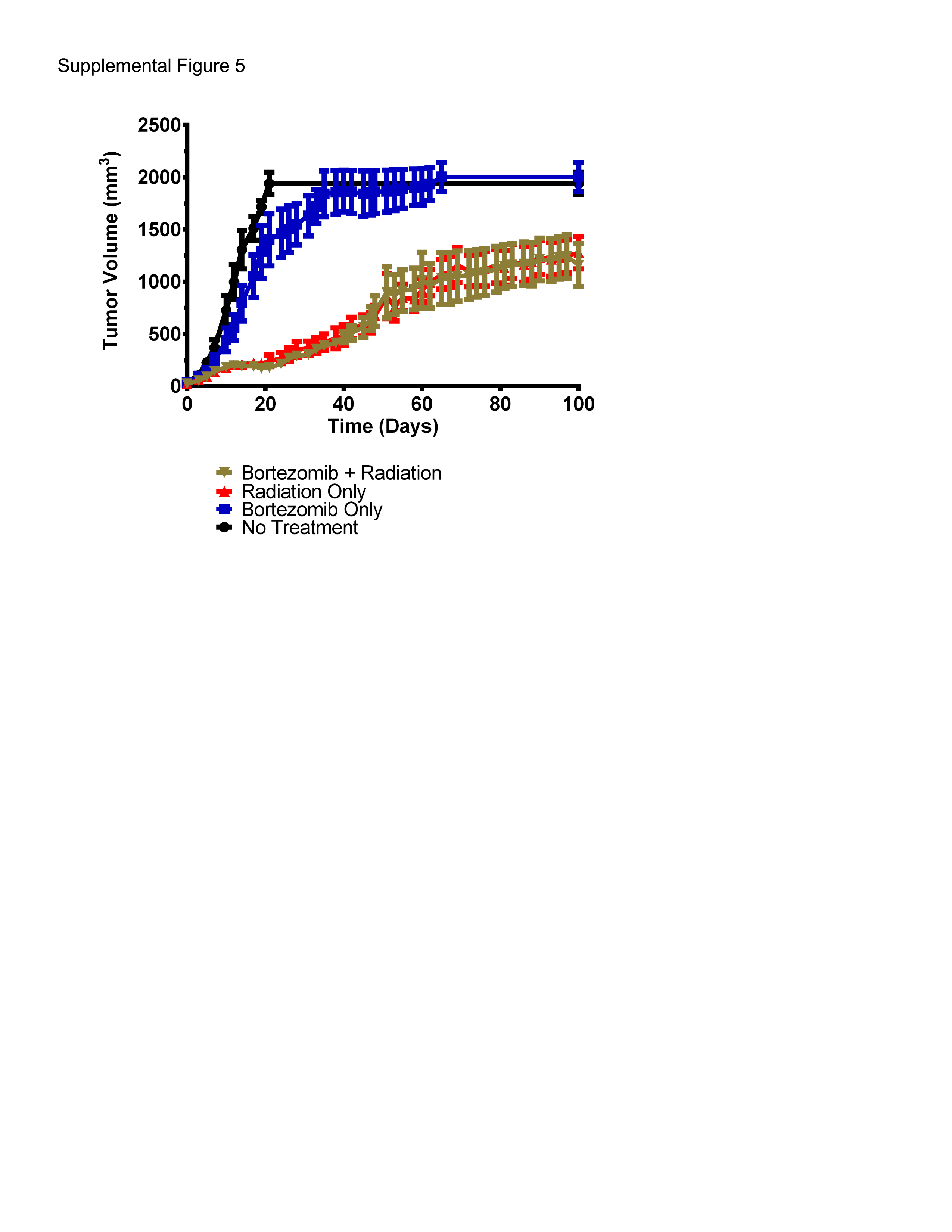

Supplement: Figure S5 — Bortezomib does not improve control of irradiated NSCLC xenografts. 106 NCI-H460 cells were injected into the flanks of 6–8 week-old NCr nude mice. Once tumors reached 3 mm diameter (day 0), bortezomib was injected into the tail vein at 0.6 mg/kg twice per week. One week later, RT was initiated to give a total of five 4 Gy fractions every other day using a small animal radiation research platform (SARRP) with 0.6 mg/kg bortezomib given 1 hour prior to RT. Mice were subsequently followed until tumors reached 2 cm diameter, animals became moribund or for 100 days. (TIF) [file pone.0073710.s005.tif]

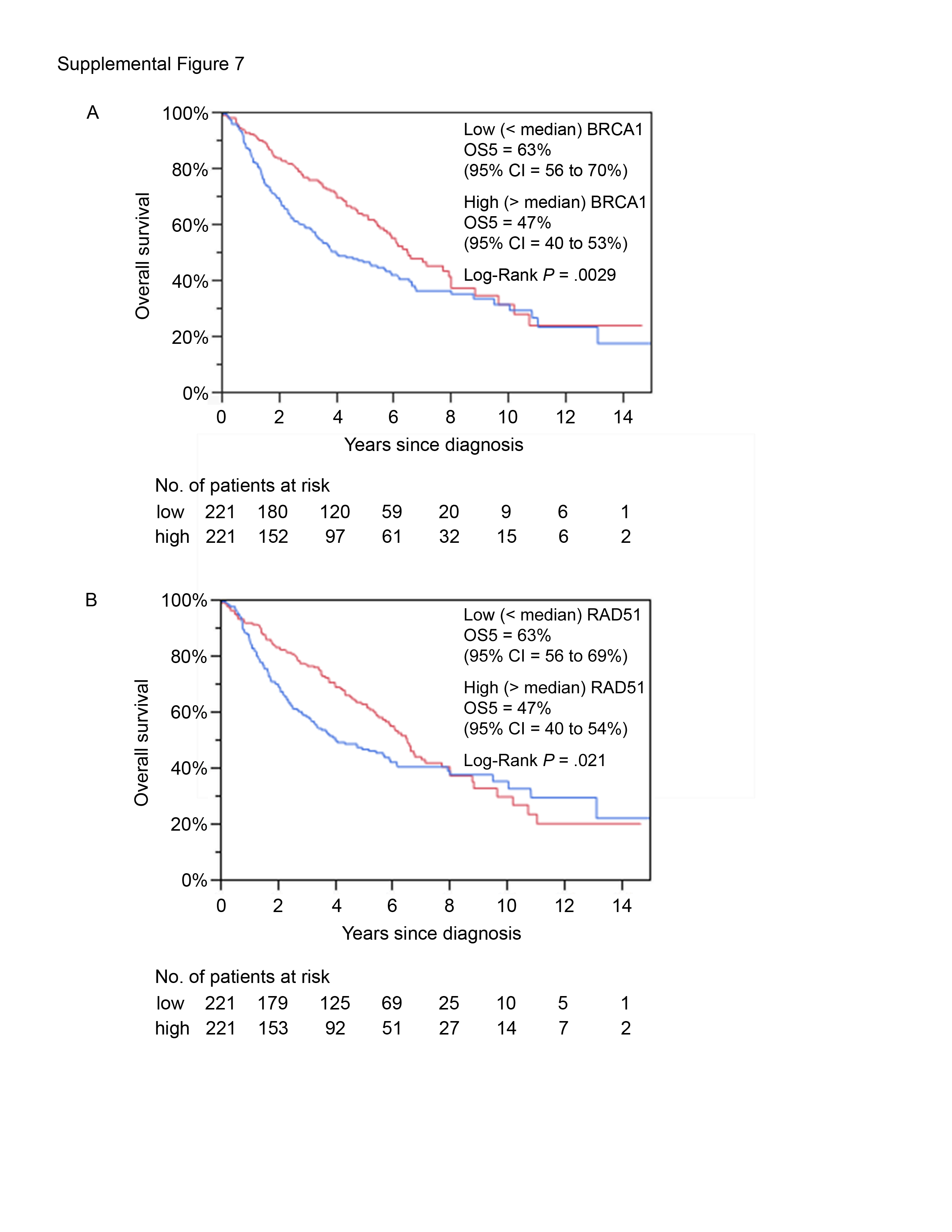

Supplement: Figure S7 — High BRCA1 and RAD51 gene expression are each associated with decreased overall survival. Kaplan-Meier analysis was performed using a published dataset of 442 lung adenocarcinomas [9], comparing survival among patients with greater than median expression versus less than median expression of each gene. The log-rank test was performed to assess for statistically significant differences in overall survival. (TIF) [file pone.0073710.s007.tif]
